# Natural Killer Cells in Uterine Adenomyosis: Immunological Mechanisms and Therapeutic Perspectives

**DOI:** 10.1155/jimr/3833044

**Published:** 2026-08-03

**Authors:** Dilimulati Yalikun, Lulu Si, Hanlin Fu, Ruixia Guo

**Affiliations:** ^1^ Department of Obstetrics and Gynecology, The First Affiliated Hospital of Zhengzhou University, Zhengzhou City 450052, Henan Province, China, zzu.edu.cn

**Keywords:** adenomyosis, fertility, immune microenvironment, immunotherapy, mechanism, natural killer cells

## Abstract

Uterine adenomyosis (AM), a common gynecological disorder, is characterized by the invasion of endometrial glands and stroma into the myometrium, resulting in chronic pelvic inflammation and severe pain. This condition not only adversely affects patients’ quality of life but also correlates with diminished fertility. Recent findings underscore the critical role of natural killer (NK) cells in the pathophysiology of AM. As a vital component of innate immunity, NK cells are responsible for identifying and eliminating abnormal cells, and functional alterations of NK cells may contribute to the progression of this disease. Although existing research has suggested preliminary associations between NK‐cell dysfunction and AM, the exact mechanistic pathways and their clinical implications are still not fully elucidated. This review comprehensively explores the involvement of NK cells in AM, emphasizing their functional adaptability within the immune microenvironment, interactions with cytokine networks, and potential for therapeutic targeting. Furthermore, we critically assess the translational hurdles and propose a strategic framework for implementing NK cell‐based immunomodulatory approaches in clinical practice.

## 1. Introduction

Uterine adenomyosis (AM), a nonneoplastic gynecological disorder, is prevalent among 20% to 35% of women of reproductive age [1], with incidence rates escalating to 60% in hysterectomy specimens from women experiencing pelvic pain [2]. The prevalence of AM has been rising in recent years, showing a noticeable trend towards younger patients [3]. Studies indicate that ~30% to 35% of individuals with AM present with symptoms, primarily affecting those aged between 30–50 years [4]. Characterized by the invasion of endometrial tissue into the myometrium, AM manifests clinically through irregular menstruation, dysmenorrhea, and infertility [5]. This condition exhibits a close pathological association with endometriosis, a syndrome defined by the existence of ectopic endometrial tissue outside the uterine cavity, which is linked to severe dysmenorrhea, chronic pelvic pain, infertility, and pelvic lesions [6]. Nevertheless, one‐third of women with AM or endometriosis remain asymptomatic [7]. Similar to endometriosis, AM is an estrogen‐dependent disease in which ectopic endometrial epithelial cells and stromal fibroblasts invade the myometrium as continuations of the normal endometrium [8].

AM not only severely impairs female fertility but also significantly reduces the quality of life, imposing considerable psychological distress and physical challenges on affected individuals [9]. The clinical management of this condition remains a formidable therapeutic challenge due to difficulties in diagnosis and limited treatment options. Current pharmacological treatments primarily provide temporary relief of symptoms and do not offer definitive agents that target the underlying pathological mechanisms. Although total hysterectomy represents a definitive surgical solution for the complete removal of lesions, this invasive procedure carries inherent risks of perioperative complications, premature ovarian insufficiency, and alterations to the pelvic floor; these may lead to irreversible effects on reproductive health and quality of life. This clinical impasse highlights the urgent need for innovative targeted therapeutic strategies that enhance patient outcomes while maintaining organ functionality [10]. Despite the advancements in research, the precise etiology of AM remains incompletely understood. Current evidence suggests a multifactorial contribution, including genetic susceptibility, hormonal imbalances, chronic inflammation, and abnormal immune responses [11].

Immune modulation in AM has been a focal point in recent research. Notably, the impact of immune dysregulation, particularly dysfunction in natural killer (NK) cells, has attracted considerable attention regarding the onset and progression of AM [12]. As an important component of innate immunity, NK cells primarily perform immune surveillance against tumor cells and virus‐infected cells [13]. Thus, they play a vital role in antitumor immunity. Recent investigations indicate that NK‐cell dysfunction may lead to immune tolerance within the endometrial microenvironment, which could facilitate the progression of AM [14, 15]. For example, studies have illustrated that diminished NK cell cytotoxicity and cytokine secretion are associated with enhanced endometrial cell proliferation, immune evasion, and disease progression [16, 17]. Furthermore, the aberrant interactions between NK cells and other local immune cell populations, such as macrophages, T cells, and dendritic cells (DCs), might aggravate chronic inflammation and tissue remodeling associated with this disease [18].

However, despite the limited systematic investigations into the mechanisms of NK cells in AM, preliminary evidence suggests their involvement through various pathways: (1) the modulation of local immune homeostasis via perforin/granzyme secretion and death receptor signaling [17]; (2) the influence on endometrial cell proliferation and apoptosis through cytokine networks, such as interferon‐gamma (IFN‐γ) and transforming growth factor‐beta (TGF‐β) [19]; and (3) the regulation of angiogenesis and neurogenesis in ectopic lesions [20]. This review aims to consolidate current understanding of the dual roles of NK cells—both protective and pathogenic—in AM and to evaluate their potential as therapeutic targets or diagnostic biomarkers, thereby providing novel insights for future research endeavors.

A comprehensive literature search was conducted across PubMed, Web of Science, and Scopus to identify relevant studies published from January 2000 to December 2025. The following search terms were used in various combinations using Boolean operators (AND/OR): “adenomyosis,” “endometriosis,” “natural killer cells,” “NK cells,” “uterine NK cells,” “immune microenvironment,” “immune dysregulation,” “immunotherapy,” “NK‐cell exhaustion,” “perforin,” “granzyme,” “cytokine,” “TGF‐beta,” “estrogen,” “hormone,” and “infertility.” Additional targeted searches were performed for specific subtopics, including “NK‐cell receptors,” “CAR‐NK therapy,” “IL‐15,” “nanoparticle delivery,” and “NK‐cell clinical trials.” Reference lists of retrieved articles and relevant review papers were also screened to identify additional eligible studies. Given the narrative nature of this review, no formal quality assessment or quantitative synthesis was performed. Studies were selected based on their relevance to the immunological mechanisms of NK cells in the context of AM, with particular emphasis on original research, authoritative reviews, and translational studies. Non‐English publications, conference abstracts without full‐text availability, and studies focusing exclusively on nongynecological malignancies without immunological mechanistic insights were excluded.

## 2. Pathogenesis and Histopathology

### 2.1. Etiology and Pathogenesis

**Figure 1:**
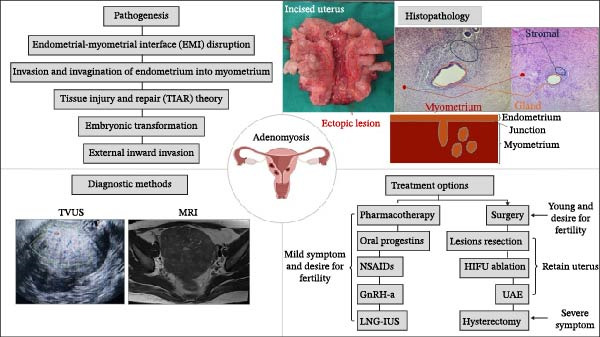
Summary of adenomyosis, the pathogenesis, histopathology, diagnosis, and treatment options. NSAIDs, nonsteroidal anti‐inflammatory drugs; GnRH‐a, gonadotropin‐releasing hormone agonist; LNG‐IUS, levonorgestrel‐releasing intrauterine system; HIFU, high intensity focused ultrasound; UAE, uterine artery embolization; TVUS, transvaginal ultrasound; MRI, magnetic resonance imaging.

The underlying causes and development mechanisms of AM are intricate and involve multiple interrelated factors, and these mechanisms remain incompletely elucidated (Figure 1). The previously proposed tissue injury and repair (TIAR) hypothesis indicated that AM and endometriosis may represent two manifestations of a singular disease [21]; however, this concept has not been thoroughly delineated in the literature. Presently, the predominant mechanisms implicated in AM encompass the infiltration of the endometrial basal layer into the myometrium [22], alterations in embryonic origin transformation, and external inward invasion of endometrial cells [23, 24]. These processes are characterized by modifications in cell behavior, hormonal activity, immunological responses, and genetic predispositions. The hypothesis regarding the invasion of the endometrial basal layer posits that factors that facilitate cell migration, proliferation, and the epithelial‐to‐mesenchymal transition (EMT) permit endometrial cells to penetrate the myometrium [25, 26]. The fundamental mechanisms governing EMT in AM still need to be further investigated. The microtrauma theory asserted that actions such as uterine contractions generate microtraumas at the interface between the endometrium and myometrium, thereby fostering further endometrial invasion through TIAR mechanisms [27, 28]. Additionally, the novel embryonic transformation theory highlights that AM may arise from residual embryonic epithelial progenitor cells or from the transformation or differentiation of adult endometrial stem cells [29]. Furthermore, the external inward invasion theory suggests that retrograde menstruation transports endometrial stem cells into the myometrium, subsequently leading to the formation of AM lesions, which may be correlated with the presence of deep endometriotic nodules [30, 31]. Research has indicated that myometrial fibrosis plays a significant role in the pathogenesis of AM [32–34], suggesting its importance in the disease progression. In addition, processes such as epithelial‐to‐endothelial transition (EET) and vasculogenic mimicry (an endothelial cell‐independent mechanism of angiogenesis) are also observed in AM [35].

Endometriosis and AM are recognized as two closely related conditions, with a notable increase in the occurrence of AM among women diagnosed with endometriosis [5, 36, 37]. Prior research indicates that AM may potentially develop from Pelvic endometriosis [38, 39]. Nonetheless, ambiguity remains concerning the precise etiology of both endometriosis and AM, and the question of whether they constitute different phenotypes of a singular disease continues to be debated among researchers. Furthermore, the pathogenesis of these conditions is intricately linked to hormonal irregularities [40], including elevated estrogen levels and progesterone resistance [41]; immune system dysfunction and immune cell activation [42, 43]; and altered expression of inflammatory mediators. Additionally, genetic factors, such as polymorphisms and epigenetic influences like aberrant DNA methylation patterns, play a role in this complex interplay [44]. Taken together, these factors significantly contribute to the development of both AM and endometriosis.

### 2.2. Histological and Diagnostic Highlights

AM is pathologically defined by the benign invasion of endometrial glands and stroma into the myometrium, typically accompanied by reactive hyperplasia of the surrounding smooth muscle [45]. Microscopic examination reveals ectopic nonneoplastic endometrial glands and stroma encompassed by a hyperplastic and thickened myometrium [46, 47]. AM, a benign gynecological condition, involves abnormal endometrial gland and stroma proliferation within the myometrium, forming nodular clusters with morphological changes due to compression [48]. These changes include irregular gland shapes and sizes, inflammation, and fibrosis. Clinically, it is classified as diffuse or focal based on imaging [49, 50]. Diffuse AM causes generalized proliferation and uterine enlargement [51]. While focal types include adenomyoma and cystic AM, where local growth resembles myomas. Histological features may correlate with symptom severity [52], differing between superficial and deep infiltrative forms. Pathology is also linked to age, hormonal status, and reproductive history [48]. In recent years, advancements in imaging technology, such as MRI and ultrasound, have become essential in the diagnosis of AM. These modalities facilitate clinicians in acquiring a deeper understanding of the histological features of the lesions and in developing effective treatment strategies.

The diagnostic process relies on clinical symptoms, menstrual history, and imaging results [53, 54]. Ultrasound and MRI are widely used, but definitive diagnosis is through pathological examination [10], the gold standard. Transvaginal ultrasound (TVUS) observes uterine morphology and myometrial thickness, while MRI provides clearer tissue images to differentiate conditions [55]. The MUSA group defined ultrasound descriptions of AM, distinguishing direct and indirect features [56, 57]. Emerging three‐dimensional ultrasound aids in early detection [58]. Moreover, as the in‐depth study of the pathophysiological mechanisms of AM progresses, new imaging biomarkers and scoring systems are continually proposed and increasingly applied in clinical practice to improve the accuracy and sensitivity of diagnosis [59]. In conclusion, delineating clinical symptoms and diagnostic criteria for AM establishes a critical foundation for early identification and subsequent therapeutic intervention.

### 2.3. Current Management and Limitations

Presently, the management of AM mainly includes pharmacological treatments and surgical intervention. For patients exhibiting mild symptoms who wish to maintain fertility, pharmacotherapy is frequently regarded as the preferred initial approach [60]. This includes the administration of nonsteroidal anti‐inflammatory drugs (NSAIDs), oral contraceptives, oral progestins, gonadotropin‐releasing hormone agonists (GnRH‐a), and the levonorgestrel intrauterine release systems (LNG‐IUS) [61]. While effective for symptom control, these treatments often fail to address the underlying immune dysregulation. Surgical options, ranging from lesion excision to total hysterectomy, offer relief but carry the risks of recurrence or loss of fertility. This clinical gap necessitates a shift toward targeted immunomodulatory strategies that can restore local uterine homeostasis without invasive interventions.

In recent years, immunotherapy has emerged as a pioneering strategy in the realm of comprehensive treatments. The fundamental concept of immunotherapy involves modulating the immune response to enhance pathological conditions, alleviate symptoms, and inhibit disease progression [62]. Given the central role of chronic inflammation and local immune dysfunction in AM pathogenesis [63], immunomodulatory approaches have gained increasing attention as potential therapeutic strategies. Increasing attention has been directed towards the involvement of NK cells in the immunotherapy of AM as their dysfunction is linked to disease advancement.

## 3. NK‐Cell Biology: Relevance to AM

### 3.1. Origin, Types, and Distribution of NK Cells

NK cells primarily originate from hematopoietic stem cells located in the bone marrow [64]. NK cells account for ~1% of the immune cell population and about 2% of lymphocytes within the human body [65]. Their distribution is predominantly observed in the liver, bone marrow, and bloodstream, and their functionality is regulated by a variety of inhibitory and activating receptors [66]. Through a succession of developmental phases, these cells differentiate into functionally active NK cells; this process is significantly influenced by various cytokines, particularly interleukin‐15 (IL‐15) and IL‐2, which are essential for the proliferation and differentiation of NK cells [67, 68].

NK cells can be categorized into distinct types based on variations in their phenotypes, functional capabilities, and tissue localization, each contributing critically to specific immune responses. In humans, NK cells are primarily divided into two major subsets: CD56^bright^ NK cells and CD56^dim^ NK cells [69], differentiated by the expression levels of CD56 and CD16 surface molecules [70, 71]. CD56^bright^ NK cells are characterized by high expression of CD56 and low expression of CD16, predominantly residing in lymphoid tissues and playing a key role in regulatory immune responses, exhibiting strong immunomodulatory properties but relatively limited cytotoxicity [72]. Conversely, CD56^dim^ NK cells, which constitute over 90% of NK cells in peripheral blood [73], are marked by low levels of CD56 and high levels of CD16 and are primarily characterized by their cytotoxic functions, including the secretion of granzymes and perforin, as well as mediating antibody‐dependent cell‐mediated cytotoxicity (ADCC) to eliminate target cells [69].

Additionally, NK cells can be further classified into additional subtypes according to the expression of their surface receptors, highlighting their increasing heterogeneity. Recent single‐cell RNA sequencing (scRNA‐seq) analyses have unveiled the diversity within NK‐cell populations, identifying major groups such as NK1, NK2, and NK3, each with distinct gene expression patterns, surface markers, cell functions, and tissue distributions [74]. For instance, NK1 cells correspond to CD56^dim^ NK cells and are primarily concentrated in the bone marrow, spleen, and blood, whereas NK2 cells align with CD56^bright^ NK cells, exhibiting higher prevalence in the lungs, tonsils, lymph nodes, and intestines [75]. The third subset, NK3 cells, mainly comprises CD16^dim^ adaptive NK cells and also possesses memory‐like capabilities, facilitating the modulation of innate immune responses [76]. Mature NK cells primarily reside in peripheral blood, spleen, liver, and lymph nodes [77]. These classification frameworks provide valuable insights into the roles of NK cells across diverse physiological and pathological conditions.

### 3.2. Basic Functions of NK Cells

NK cells serve as important effector components of the innate immune system, primarily responsible for the surveillance and elimination of neoplastic cells and virus‐infected cells [78]. Through their unique recognition mechanisms, NK cells can quickly detect and eradicate transformed or infected cells independently of antigen‐presenting cells and antibodies [79]. They assess the expression level of molecules such as major histocompatibility complex (MHC) class I through specific inhibitory receptors [80]. Research has indicated considerable differences in the proportions and functional profiles of NK cells across different tissues. For example, NK cells residing in the liver demonstrate strong immunoregulatory capabilities. In contrast, those in peripheral blood are more focused on cytotoxic activities [81]. However, the functions of NK cells are not limited to cytotoxicity. They also secrete cytokines to modulate the activity of other immune cells, thereby exerting multiple roles in immune regulation [82]. Their function mainly relies on the activation status of the surface receptors.

**Figure 2:**
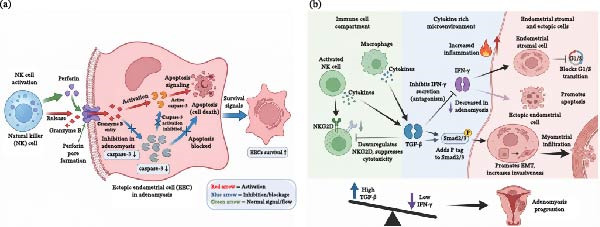
Dual regulatory pathways govern EECs’ fate in adenomyosis. (a) Molecular mechanism of perforin/granzyme pathway‐induced apoptosis in adenomyosis. Perforin forms transmembrane pores to facilitate granzyme B entry, which activates the caspase‐3 and triggers EEC apoptosis; this pathway is inhibited in adenomyosis, leading to EEC survival. Red arrows indicate activation, blue arrows indicate inhibition or blockage, and green arrows represent normal signaling or flow. (b) TGF‐β cytokine network: NK cell‐secreted IFN‐γ blocks cell cycle G1/S transition to inhibit EEC proliferation and promote apoptosis, while TGF‐β downregulates NKG2D to suppress NK‐cell cytotoxicity and activates the Smad2/3 pathway to induce EMT, enhancing EECs’ invasiveness and myometrial infiltration. TGF‐β antagonizes inflammation, disrupts cytokine balance, and accelerates adenomyosis progression.

The mechanisms governing the activation and inhibition of NK cells are crucial for their functional efficacy. NK cells express various activating and inhibitory receptors on their cell surfaces, which determine their activation state by recognizing specific molecules on the surfaces of target cells. Activating receptors on NK cells, such as NKG2D and NKp46, are capable of detecting stress‐induced molecules present on tumor cells or infected cells, thereby initiating cytotoxic responses [83]. Simultaneously, NK‐cell activity is modulated by inhibitory signals, primarily through inhibitory receptors, including killer immunoglobulin‐like receptors (KIRs), which interact with MHC class I molecules on the surfaces of target cells [84]. Typically, normal cells express MHC class I molecules, whereas tumor cells and infected cells frequently lack MHC class I expression [85]. This inhibitory mechanism plays a critical role in ensuring that NK cells preferentially target abnormal cells while protecting normal cells from self‐attacks [86]. Additionally, various factors present in the tumor microenvironment, such as cytokines and metabolites released by tumor cells, can significantly impact the activation state of NK cells, leading to functional impairment, thereby aiding in the immune evasion of tumor cells [87]. In such microenvironments, there is often an upregulation of inhibitory receptors on NK cells and an increased expression of their ligands on target cells, coupled with a downregulation of activating receptors on NK cells and their corresponding ligands on target cells; this results in compromised NK‐cell functions [88]. Consequently, a comprehensive understanding of the activation and inhibition mechanisms governing NK cells is essential for the advancement of innovative immunotherapeutic approaches. Specifically, the perforin/granzyme pathway (Figure 2a) is a key effector mechanism by which NK cells mediate the apoptosis of ectopic endometrial cells (EECs) in AM. Upon activation, NK cells release perforin, which forms transmembrane pores on the surface of target endometrial cells, enabling granzyme (primarily granzyme B) to enter the cytoplasm. Granzyme B then activates the caspase‐dependent apoptotic pathway, specifically cleaving caspase‐3 and caspase‐9, which in turn triggers the degradation of cellular proteins and eventual apoptosis of EECs [89]; analogous perforin/granzyme‐mediated killing has also been documented in endometriosis‐associated NK‐cell dysfunction [90]. However, in patients with AM, the expression and secretion of perforin and granzyme B in NK cells are significantly downregulated [91], as observed in the related condition of endometriosis [92], likely contributing to the failure of effective elimination of EECs, which further proliferate and invade the myometrium.

### 3.3. Differential Alterations of Uterine NK (uNK) Cells and Peripheral NK (pNK) Cells

It is imperative to clearly distinguish between uNK cells and pNK cells as they possess distinct physiological origins, phenotypes, and functions [91]. Physiologically, pNK cells are predominantly cytotoxic (CD56^dim^CD16+) and tasked with systemic immunosurveillance. In contrast, uNK cells are a highly specialized, tissue‐resident population (predominantly CD56^bright^CD16−) whose primary physiological role is not cytotoxicity but rather orchestrating spiral artery remodeling, angiogenesis, and decidualization to prepare for pregnancy [92].

In the context of AM, these two distinct populations are affected in fundamentally different ways. The alteration of pNK cells primarily manifests as a generalized systemic suppression of their cytotoxic capacity, rendering them less effective at clearing disseminating endometrial cells in the circulation [63]. Conversely, specialized uNK cells undergo a pathological functional shift within the local microenvironment. Because their inherent physiological nature is proangiogenic and immunomodulatory, uNK cells are more easily “hijacked” by the adenomyotic milieu [70]. Instead of supporting normal endometrial cyclic shedding and pregnancy, these altered uNK cells inappropriately secrete excessive levels of vascular endothelial growth factor (VEGF) and matrix metalloproteinases (MMPs). This hijacked uNK population directly fuels aberrant angiogenesis, tissue remodeling, and the survival of ectopic endometrial glands deep within the myometrium [93].

## 4. The Role of NK Cells in AM

### 4.1. NK‐Cell Dysfunction in AM

In patients diagnosed with AM, the term “NK‐cell dysfunction” encompasses a complex interplay of numerical alterations, phenotypic shifts, and severe functional exhaustion, which exhibits significant spatial heterogeneity between the peripheral blood and the local uterine microenvironment. Regarding cell numbers vs. function, current literature presents a prevailing view that the primary defect in AM lies in the profound impairment of NK‐cell cytotoxicity rather than uniform declines in absolute numbers [17]. However, a critical examination of the current literature reveals notable discrepancies and conflicting data regarding the exact nature of NK‐cell alterations, particularly whether the disease is primarily driven by a numerical deficiency or a functional impairment. Some studies report a moderate decrease in the absolute count of NK cells in the peripheral blood and endometrium of AM patients [16], suggesting a systemic and local depletion of immune effectors. Conversely, other researchers have found no significant numerical differences but highlight a drastic reduction in their cytotoxic capabilities, as demonstrated in the related disease endometriosis [90], where stromal cell‐macrophage crosstalk impairs NK‐cell cytotoxicity. Whether this functional impairment operates identically in AM remains to be directly confirmed. This functional impairment is characterized by a significant downregulation of activating receptors (such as NKG2D and NKp30) and a diminished capacity to secrete perforin and granzyme B, a phenotype consistent with the general features of NK‐cell exhaustion described in the tumor microenvironment [88], rendering them incapable of eliminating EECs.

These observed discrepancies in NK‐cell abundance and activity can be attributed to several critical biological and methodological factors [65], and similar methodological considerations have been raised in endometriosis research [92]. First, spatiotemporal heterogeneity plays a decisive role; uNK cells undergo dynamic fluctuations driven by steroid hormones across the menstrual cycle, and studies that do not strictly stratify patients by cycle phase often yield conflicting results [70, 92]. Second, tissue‐specific microenvironments differ significantly, where the immunological milieu of ectopic lesions is far more immunosuppressive than that of peripheral blood or the eutopic endometrium [18]. Notably, analogous findings in endometriosis demonstrate that the lesional microenvironment drives NK‐cell exhaustion through programmed cell death protein 1 (PD‐1) upregulation [94], a mechanism that likely extends to AM. Third, methodological variations in flow cytometry gating—such as the broad use of CD56 without differentiating between the proangiogenic CD56^bright^ and cytotoxic CD56^dim^ subsets—can mask the true functional shift occurring in AM [71, 74]. Consequently, while numerical counts remain debated, the consensus points toward a profound functional exhaustion within the localized lesion site.

Furthermore, the alterations of NK cells strictly depend on their spatial localization. The pNK cells in AM patients often display a systemic but milder state of immune suppression. In stark contrast, within the local uterine microenvironment (both eutopic endometrium and ectopic myometrial lesions), NK cells are subjected to a highly immunosuppressive and hyperestrogenic niche [40]. In this local peritoneal and myometrial environment, the chemotactic movement of NK cells is substantially hindered, a phenomenon well‐documented in pelvic endometriosis [95] and likely operative in AM, and their cytotoxic activity is heavily suppressed by local factors such as TGF‐β, IL‐10, and prostaglandin E2 (PGE2). In endometriosis, PGE2 is recognized as a master regulator of immune evasion [94], given the shared inflammatory milieu, it is plausible that PGE2 exerts similar immunosuppressive effects in AM, leading to a state of profound cellular exhaustion, as characterized in advanced endometriosis [96] and increasingly recognized in AM.

Crucially, the disease‐specific dysfunction in AM is characterized by a shifted balance between NK‐cell subsets, specifically the CD56^bright^ and CD56^dim^ populations. In a healthy reproductive tract, uNK cells are predominantly of the CD56^bright^CD16‐ phenotype, playing a regulatory role in angiogenesis and tissue remodeling, while pNK cells are predominantly the cytotoxic CD56^dim^CD16+ subset [71, 72]. In the context of AM, this subset balance is skewed. The cytotoxic CD56^dim^ subset becomes functionally exhausted and fails to execute immunosurveillance against invading endometrial cells [97]; similar exhaustion of the CD56^dim^ compartment has been documented in endometriosis, where IL‐15 mediated stromal cell proliferation further compromises NK‐cell cytotoxicity [91]. Conversely, the CD56^bright^ subset, which is physiologically prone to secreting proangiogenic and immunomodulatory factors, may be paradoxically hijacked by the AM microenvironment [76]. Instead of maintaining normal cyclic homeostasis, these regulatory CD56^bright^ cells may inadvertently promote ectopic vascularization [98] (via VEGF secretion) and induce immune tolerance, thereby facilitating the survival, deep infiltration, and progression of adenomyotic lesions.

Research has found that the expression levels of inhibitory receptors on the surface of NK cells in the endometrium of women with reproductive disorders are significantly elevated, including activating receptors such as NKp46 [99], a finding that warrants dedicated investigation in AM patients specifically. This increased expression subjects NK cells to persistent inhibitory signals, further impairing their function and reducing their cytotoxic activity and cytokine secretion capacity, a paradigm well‐established in tumor immunology [100, 101] and plausibly operative in AM. Consequently, this contributes to a state of NK‐cell exhaustion, mirroring the PD‐1+ exhausted NK‐cell phenotype identified in advanced endometriosis [96], impairing immune responses and hindering the removal of AM‐related pathological cells, which may promote the development and progression of AM. Therefore, changes in both the quantity and activity of NK cells not only characterize the immune profile associated with AM but also provide a foundation for developing targeted immunotherapeutic strategies, such as enhancing NK‐cell cytotoxicity or blocking inhibitory receptor pathways.

Collectively, Figure 2 illustrates a synergistic mechanism of immune evasion in AM. While the suppression of the perforin/granzyme pathway leads to a “failed execution” of invading cells, the hyperactivation of the TGF‐β network creates a “protective shield.” This dual‐layer disruption not only prevents the elimination of EECs but also actively promotes their invasiveness via EMT, driving the progression of adenomyotic lesions deeper into the myometrium.

A detailed synthesis of the principal studies investigating NK‐cell alterations in AM and related conditions, including the study design, sample characteristics, and reported discrepancies, is provided in Table S1.

### 4.2. The Relationship Between NK Cell‐Mediated Immune Responses and AM

**Figure 3:**
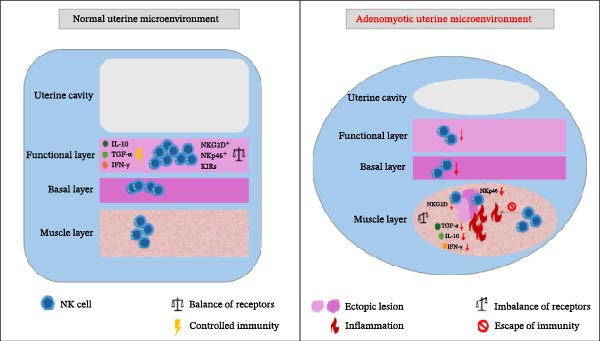
Comparison of NK cells in the microenvironment of a normal uterus and adenomyosis. Left panel: Normal uterine NK (uNK) cells with balanced cytotoxicity/cytokines, maintaining immune homeostasis. Right panel: Adenomyosis‐associated NK‐cell depletion, impaired killing (NKG2D/NKp30↓), and proinflammatory cytokine shift (TNF‐α/IL‐6↑, IL‐10/TGF‐β↓), driving ectopic lesion survival and progression.

NK cells are integral to the immune microenvironment associated with AM, where they regulate local immune responses through the secretion of various cytokines and inflammatory mediators (Figure 3). However, as the disease advances, the interplay between local inflammatory factors and immune cells can alter NK‐cell functionality, resulting in reduced effectiveness. Within the adenomyotic microenvironment, the regulation of NK‐cell activity may lead to a decreased release of inflammatory mediators, such as tumor necrosis factor‐alpha (TNF‐α), IL‐6, IL‐10, and IFN‐γ, a cytokine pattern reminiscent of the impaired NK‐cell function observed in pelvic endometriosis [95]. Among them, the TGF‐β/IFN‐γ cytokine network plays a pivotal role in regulating the proliferation and apoptosis of endometrial cells (Figure 2b). IFN‐γ, secreted by activated NK cells, can inhibit the proliferation of endometrial stromal cells by blocking the G1/S phase transition of the cell cycle and promote the apoptosis of EECs [102]. Such IFN‐γ‐mediated apoptosis of endometriotic cells has been experimentally demonstrated in endometriosis models [103]; however, in patients with AM, the secretion of IFN‐γ by NK cells is significantly reduced, and this inhibitory effect is weakened or even lost [104]. In contrast, TGF‐β is highly expressed in the adenomyotic microenvironment and mainly secreted by abnormally activated NK cells and macrophages. In the related condition of endometriosis, elevated TGF‐β derived from the lesional microenvironment drives NK‐cell dysfunction and promotes disease progression [102], and a similar mechanism may operate in AM. TGF‐β not only inhibits the cytotoxicity of NK cells by downregulating the expression of activating receptors such as NKG2D but also promotes the EMT of endometrial cells through the Smad2/3 signaling pathway, enhancing their invasiveness and ability to infiltrate the myometrium [103]. Furthermore, TGF‐β and IFN‐γ exhibit an antagonistic relationship in the adenomyotic microenvironment: the high expression of TGF‐β further suppresses the secretion of IFN‐γ by NK cells, breaking the balance of the cytokine network and accelerating the progression of AM. They not only enhance local inflammatory responses but may also influence endometrial remodeling and overall reproductive function.

### 4.3. Etiology and Reversibility of NK‐Cell Dysfunction

A critical question in the immunopathology of AM is whether NK‐cell dysfunction represents a stable genetic defect or a reversible state of exhaustion. Current evidence leans toward the latter, suggesting that NK cells in AM undergo functional exhaustion rather than suffering from permanent lineage impairment. In advanced endometriosis, a comparable state of exhaustion marked by PD‐1 upregulation has been characterized [94], and similar immunomodulatory mechanisms involving TGF‐β‐driven dysfunction have been reported in endometriosis models [105]. This state is characterized by the high expression of immune checkpoint molecules (e.g., PD‐1 and TIGIT) and can be partially reversed in vitro through stimulation with activating cytokines such as IL‐15 or IL‐2, as demonstrated using endometriosis‐derived NK cells [92] and in other ex vivo systems [106]. This reversibility indicates that the “dysfunction” is a plastic state maintained by the oppressive local microenvironment. However, it is important to note that these rescue experiments have been conducted exclusively in vitro ex vivo; whether endogenous NK‐cell function can be similarly restored in AM patients in vivo remains to be demonstrated through clinical studies.

The etiology of this exhaustion is multifactorial, driven by a “vicious cycle” of hormonal imbalance and chronic inflammation. AM is an estrogen‐dependent and progesterone‐resistant disease. High local levels of 17β‐estradiol (E2) directly impair NK‐cell activity. E2 has been shown to downregulate the expression of the activating receptor NKG2D and diminish the production of IFN‐γ via estrogen receptor‐alpha (ERα) signaling [40, 104]. Furthermore, progesterone resistance in the AM endometrium disrupts the normal physiological maturation of uNK cells, which is typically regulated by progesterone‐induced cytokines. Ectopic lesions are sites of intense chronic inflammation. Elevated levels of PGE2 and TGF‐β, which are key features of the AM microenvironment [65], act as potent inhibitors of NK‐cell metabolism and effector function. In endometriosis, TGF‐β has been shown to exacerbate NK‐cell dysfunction and impair reproductive outcomes [105], underscoring the therapeutic relevance of targeting this pathway in related uterine disorders. PGE2, in particular, signals through EP2/EP4 receptors on NK cells to increase intracellular cAMP, which serves as a molecular “brake” on their cytotoxic machinery.

In summary, NK‐cell dysfunction in AM is a reversible state of exhaustion primarily driven by the synergistic effects of a hyperestrogenic environment and a suppressive inflammatory milieu. This understanding is pivotal for therapy as it suggests that neutralizing local suppressive factors or providing exogenous cytokine support could “reawaken” the endogenous anti‐AM immune response.

### 4.4. Disease‐Specific Mechanisms: Hormone‐Driven Immune Evasion

It is critical to distinguish the mechanisms underlying NK‐cell dysfunction in AM from those in generic inflammatory or autoimmune diseases. In classical autoimmune disorders (e.g., rheumatoid arthritis), immune cells typically exhibit aberrant hyperreactivity and destruction of normal self‐tissues. In stark contrast, AM is characterized by a unique hormone‐driven, locally immunosuppressive microenvironment that closely resembles the “immune evasion” mechanisms observed in solid tumors [105].

The disease‐specific specificity of AM is primarily rooted in local hyperestrogenism. Unlike generic inflammation, the excessively high levels of local 17β‐estradiol (E2) in the AM microenvironment directly suppress NK‐cell cytotoxicity. Estrogen signaling downregulates the expression of activating receptors (such as NKG2D) on NK cells and inhibits the release of cytolytic granules (granzymes and perforin) [5, 107].

Furthermore, unlike normal tissues undergoing generalized inflammation, the ectopic endometrial stromal cells in AM actively paralyze local NK cells. They act similarly to tumor cells by upregulating tolerogenic molecules, such as human leukocyte antigen G (HLA‐G). The binding of HLA‐G to inhibitory receptors (e.g., KIR2DL4) on NK cells directly turns off their killing capacity. Thus, the NK‐cell dysfunction in AM is not a nonspecific inflammatory exhaustion but rather a disease‐specific, estrogen‐facilitated immune tolerance that allows ectopic lesions to survive and invade the myometrium [106].

## 5. Interactions Between NK Cells and Other Immune Cells

**Figure 4:**
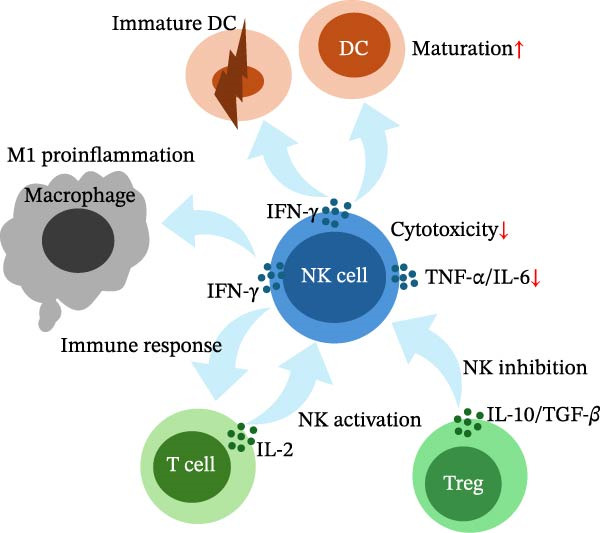
Dysfunctional NK‐cell activity (reduced cytotoxicity, skewed cytokines) disrupts immune surveillance, promotes proinflammatory macrophage polarization, and fails to counteract Treg‐mediated suppression, collectively enabling ectopic lesion persistence.

NK cells are not only capable of performing independent immune roles but also regulate the overall immune response through their engagement with various immune cells. In the case of AM, the interactions between NK cells and DCs, macrophages, and T cells are particularly significant [18] (Figure 4). Research has demonstrated that NK cells can enhance the maturation and activation of DC through the secretion of cytokines, thereby boosting the antigen‐presenting capabilities of DC [108]. In instances where DCs are immature, NK cells may directly eliminate these cells to avert the immune system from developing tolerance to self‐derived antigens [109]. Furthermore, NK cells can also influence the function of macrophages by direct interactions or by the secretion of cytokines, thereby regulating macrophage polarization towards M1 (proinflammatory) or M2 (anti‐inflammatory) phenotypes, which in turn affects local inflammatory responses and tissue repair mechanisms [98].

The interaction between NK cells and T cells is vital for maintaining immune homeostasis [110]. NK cells enhance T‐cell immune responses by secreting IFN‐γ while concurrently inhibiting overactive T cells through PD‐L1 interactions or direct contact, thus preventing immune dysregulation [111]. Conversely, T cells can activate NK cells by the release of IL‐2, whereas regulatory T cells (Tregs) suppress NK‐cell functions by secreting IL‐10 and TGF‐β, thereby maintaining immune balance [112]. Within the context of AM, these intricate interactions among immune cells may lead to either immune tolerance or immune dysregulation, each of which subsequently influences disease progression and fertility of affected individuals. Therefore, acquiring a more profound understanding of the mechanisms governing NK‐cell interactions with other immune components is essential for elucidating the immunopathological characteristics of AM and for developing potential therapeutic interventions.

## 6. Prospects of NK Cells as Potential Therapeutic Targets

### 6.1. Exploration of Immunotherapy Strategies: Lessons From Oncology

Translating NK cell‐based therapies from oncology to benign chronic conditions like AM requires a nuanced approach. To clearly delineate the landscape of potential interventions, we categorize these emerging strategies into four distinct modalities: cytokine‐based modulation, antibody‐mediated enhancement, adoptive cell therapy, and nanotechnology‐assisted targeted delivery.

#### 6.1.1. Cytokine‐Based Modulation

NK cells are capable of inducing direct cytotoxicity against tumor cells through the release of perforin and granzyme while simultaneously modulating adaptive immune responses by secreting various cytokines, such as IFN‐γ and TNF‐α, thus forming a multilayered antitumor immune network. Studies have demonstrated that stimulation with cytokines such as IL‐2, IL‐12, IL‐15, and IL‐21 can significantly enhance NK‐cell proliferation, activation, and effector functions [113]. For example, IL‐15 is crucial for the proliferation and survival of NK cells, promoting their cytotoxic activity through binding to IL‐15 receptors [114]. Additionally, after binding to their receptors, IL‐21 can further activate the antitumor activities of NK cells and synergize with IL‐15, providing possibilities for combination therapies [115].

#### 6.1.2. Antibody‐Mediated Enhancement

Building on the cellular mechanisms of NK cells, the utilization of monoclonal antibodies has been shown to improve the ability of NK cells to recognize target cells [116], thereby offering an important pathway for immunotherapy. For instance, ADCC is a crucial mechanism by which NK cells mediate the destruction of tumor cells [117]. Agents such as trastuzumab, targeting HER2, and rituximab, targeting CD20, have been documented to significantly enhance the cytotoxic potential of NK cells through the ADCC mechanism [118]. Additionally, these monoclonal antibodies augment the recognition of tumor cells by NK cells via distinct pathways [119].

#### 6.1.3. Nanotechnology for Precision Delivery

Moreover, the application of nanotechnology presents new ideas for targeted NK‐cell therapies, such as using nanocarriers to facilitate the delivery of antitumor agents to NK cells. This methodology not only increases the local concentration of therapeutic agents but also minimizes systemic side effects. Nanocarriers have the advantages of strong targeting, good biocompatibility, and the ability to improve the stability of therapeutic agents, making them an ideal delivery system for key cytokines that regulate the NK‐cell function. IL‐15, as a core cytokine for the proliferation, activation, and maintenance of cytotoxicity of NK cells, is significantly downregulated in patients with AM [120], which is an important reason for NK‐cell dysfunction [121]. Therefore, the delivery of IL‐15 using nanocarriers has become an important research direction for targeted activation of NK cells and has been proposed as a potential strategy for AM treatment, though this concept has not yet been tested in AM models. Studies have demonstrated that the delivery of IL‐15 via nanoparticles significantly enhances NK‐cell activation and improves tumor suppression, thereby enhancing therapeutic efficacy [122, 123]. Ongoing research is also focused on developing immunotherapeutic strategies that target NK‐cell surface activation receptors as activating these receptors has been shown to enhance the antitumor activity of NK cells, ultimately contributing to improved treatment outcomes [124]. It should be emphasized that all nanocarrier‐based IL‐15 delivery studies cited herein were conducted in an oncology model, and their safety and efficacy in the context of AM remain entirely unexplored.

#### 6.1.4. Adoptive Cell Therapy

Chimeric antigen receptor‐NK (CAR‐NK) cell therapy has emerged as a promising allogeneic strategy, offering several intrinsic advantages such as independence from HLA (human leukocyte antigen) matching and a reduced risk of graft‐versus‐host disease (GVHD) [125, 126]. Despite these advantages, the clinical implementation of this therapy faces significant challenges related to gene transfer efficiency and the persistence of the cells [127]. While the initial results regarding efficacy and tolerability are promising, the considerable variability in cell sources and treatment protocols highlights the necessity for larger, controlled trials and standardized methodologies to confirm the clinical relevance of CAR‐NK‐cell therapy.

Although NK‐cell immunotherapy has primarily focused on the field of tumor diseases, its potential application in immune‐related diseases is gradually gaining attention. In the pathological process of AM, it is postulated that abnormalities in NK‐cell function may result from alterations in the microenvironment of the affected tissues, which in turn impair NK‐cell functions. In the future, modulating the activity of NK cells to improve the immune microenvironment of patients with AM may become a new therapeutic direction.

### 6.2. Clinical Trials and Future Research Directions

Currently, clinical trials targeting NK cells are being gradually initiated. These trials mainly focus on assessing the safety and efficacy of NK‐cell therapies, with a primary focus on hematological malignancies and solid tumors [128, 129]. Future research directions may include creating new NK cell‐derived products, such as using genetic modification to enhance the antitumor capabilities of NK cells [130]. Moreover, combining NK‐cell therapy with other immunotherapeutic approaches, such as immune checkpoint inhibitors, may become important strategies to improve therapeutic efficacy; however, these combination strategies have thus far been investigated exclusively in oncology settings and have not been evaluated in benign gynecological conditions. To date, no registered clinical trials have evaluated NK cell‐based immunotherapy for AM. Published studies are limited to basic and observational research, focusing on the abnormal quantity, cytotoxicity, and effector molecule expression (perforin and granzyme B) of NK cells in patients with AM. Although preclinical evidence suggests that IL‐15 may enhance NK‐cell activity and potentially alleviate adenomyotic lesions, these findings have not been validated in human clinical trials. Through continuous clinical trials and basic research, NK cells are expected to play an increasingly important role in cancer or noncancer immunotherapy.

### 6.3. Key Unresolved Questions and Translational Challenges

Despite the growing evidence implicating NK cells in the pathogenesis of AM, several fundamental questions remain unresolved, significantly hindering clinical translation. The most critical unanswered question is the precise temporality of immune dysregulation. It remains unclear whether a preexisting, inherent defect in NK‐cell immunosurveillance permits the initial invagination and survival of endometrial cells in the myometrium or if the ectopic lesions’ activity paralyze initially healthy NK cells by establishing a hyperestrogenic, TGF‐β‐rich, and immunosuppressive microenvironment. Current cross‐sectional clinical data are insufficient to disentangle this “chicken‐and‐egg” paradigm. Future research employing longitudinal animal models, combined with lineage tracing and single‐cell transcriptomics at the very early stages of disease onset, is urgently needed to determine the exact sequence of immunological events.

A significant barrier to addressing this question is the limited repertoire of validated AM animal models. The tamoxifen‐induced neonatal mouse model, which recapitulates key histopathological features of AM, including myometrial infiltration and altered immune profiles, represents the most widely used system [16]. However, this model relies on pharmacological induction in neonatal mice, which does not fully replicate the spontaneous, hormone‐driven, and age‐dependent onset observed in human AM. Alternative approaches, such as endometrial–myometrial suture or pituitary grafting models, have been described but remain poorly characterized with respect to immune parameters and lack standardized protocols. The absence of a model that faithfully reproduces the chronic, progressive nature of human AM—including the cyclical hormonal fluctuations and the full spectrum of immune dysregulation—constitutes a major bottleneck for preclinical testing of NK cell‐directed therapies.

Furthermore, translating NK cell‐based immunotherapies from oncology to gynecology presents a unique safety paradox. In life‐threatening malignancies, aggressive systemic immune activation (e.g., via systemic high‐dose cytokines or CAR‐NK cells) is often clinically acceptable despite the high risk of severe adverse events. In stark contrast, AM is a benign, chronic condition affecting reproductive‐aged women. Systemically hyperactivating NK cells in these patients poses an unacceptable risk of inducing off‐target autoimmunity, systemic inflammatory toxicity (e.g., cytokine release syndrome), or disrupting normal maternal–fetal immune tolerance crucial for future pregnancies.

Therefore, to safely apply NK cell‐based therapies in AM without causing autoimmunity, future strategies must strictly prioritize spatial and temporal precision. We propose that research should pivot toward localized and transient immunomodulation. For instance, utilizing an intrauterine delivery system (e.g., modifying the existing LNG‐IUS to corelease low‐dose NK‐activating cytokines like IL‐15) or employing targeted nanocarriers that confine immune activation strictly within the uterine microenvironment. By limiting the therapeutic action to the local pelvic niche, it may be possible to reawaken NK‐cell immunosurveillance against ectopic lesions while safely preserving systemic immune homeostasis.

## 7. Discussion

In this review, we explored the critical role of NK cells in the pathogenesis of AM. As essential components of the innate immune system, NK cells are involved in modulating the immune response, and their dysfunction may contribute to the onset and progression of AM. Through a comprehensive synthesis and analysis of existing studies, it became evident that NK cells could affect both the clinical manifestations presented by patients and their responses to various treatments. However, inconsistencies regarding the roles and impacts of NK cells were noted across different studies, likely reflecting variations in the study design, sample selection, and experimental methods.

A fundamental unresolved question contributing to these inconsistencies is the “cause versus consequence” paradox. It remains heavily debated whether a primary systemic NK‐cell defect permits the initial invagination of endometrial glands into the myometrium or if the profound local suppression—driven by the hyperestrogenic and TGF‐β‐rich microenvironment of established lesions—secondarily “exhausts” recruited NK cells. Pinpointing the exact timing of immune evasion during disease onset is a critical hurdle that future longitudinal studies must overcome.

Currently, treatment modalities for AM—ranging from NSAIDs and GnRH‐a to surgical excision or hysterectomy—are fraught with challenges. High recurrence rates, long‐term side effects, and the devastating loss of fertility make these conventional options suboptimal for many reproductive‐aged women. While emerging interventions like uterine artery embolization offer minimally invasive alternatives, lasting symptom improvement requires further validation. Consequently, immunotherapy has emerged as a prominent strategy to tackle the core issue by precisely modulating the immune response. Correcting the abnormal immune state could alleviate symptoms like pain and menorrhagia while restoring the immune balance at the root level to prevent recurrence.

NK cells represent a highly promising target for immunotherapeutic interventions. However, the translational leap from oncology (where NK therapies are well‐established) to gynecology presents a unique safety‐efficacy tradeoff. Unlike treating lethal malignancies, managing a benign, chronic condition like AM requires strict preservation of the patient’s physiological integrity and future pregnancy tolerance. High‐quality clinical data remains lacking; no prospective, registered clinical trials have yet investigated systemic NK‐cell infusion, IL‐15 therapy, or immune checkpoint inhibitors specifically for AM. To circumvent potential systemic autoimmunity or cytokine‐related toxicities, future translational research must prioritize localized delivery systems. Strategies such as drug‐eluting intrauterine devices (IUDs), targeted nanoparticles, or ultrasound‐guided intralesional injections could gently and accurately “reawaken” uNK cells without perturbing peripheral immune homeostasis.

To bridge the gap between the bench and bedside, future research must also embrace next‐generation technologies. The application of scRNA‐seq and spatial transcriptomics is urgently needed to map the heterogeneity of NK‐cell subsets (e.g., tissue‐resident vs. peripherally recruited NK cells) within the myometrial junctional zone. Furthermore, the lack of reliable animal models has historically limited preclinical testing. The development of patient‐derived AM organoids cocultured with autologous NK cells could provide a revolutionary, patient‐specific platform to validate the efficacy and safety of NK cell‐directed strategies before clinical trials.

Therefore, it is important to maintain an open‐minded approach when interpreting different research findings while reasonably assessing the credibility and applicability of each study. Moreover, it is crucial to emphasize the importance of multidisciplinary collaboration—bridging immunology, oncology‐derived cell engineering, and gynecology—to foster a more comprehensive understanding of the relationship between NK cells and AM, ultimately bringing precision immunotherapy to clinical reality.

## 8. Conclusion

In summary, although current therapeutic strategies possess both advantages and limitations, immunotherapy stands out due to its precise targeting capability, consideration of reproductive health, and potential for sustained disease management. Extensive and in‐depth research is urgently needed to refining treatment protocols and exploring synergistic approaches that could pave the way for possible cures for patients. Future research should focus more on the regulatory mechanisms governing NK cells, particularly focusing on how modifying NK‐cell functions could improve the prognosis and quality of life for patients in the case of AM. Additionally, investigating the prospective roles of NK cells in the treatment of AM, particularly through the development of immunotherapeutic interventions, may provide new avenues for treatment. As foundational research advances and clinical applications broaden, studies involving NK cells are expected to yield novel solutions for the management of AM, thereby improving the overall health outcomes for patients. In conclusion, NK cells exhibit significant potential in the research of AM; through rigorous scientific investigation and clinical practice, we can achieve a deeper understanding of their roles in this disease, ultimately offering more effective treatment strategies for affected individuals.

## Author Contributions

Writing – original draft preparation, writing – review: Dilimulati Yalikun. Formal analysis, editing: Lulu Si. Methodology, validation: Hanlin Fu and Lulu Si. Supervision, funding acquisition: Ruixia Guo.

## Funding

This research was funded by the National Natural Science Foundation of China (NSFC) (Grant 82273229 to Ruixia Guo and Grant 82403060 to Lulu Si), the Provincial‐Ministrial Co‐constructed Key Program of Henan Province (Grant SBGJ202302074 to Ruixia Guo), and the Young Talent Support Program of Natural Science Foundation of Henan Province (Grant 242300420385 to Lulu Si).

## Disclosure

All authors have read and agreed to the published version of the manuscript.

## Ethics Statement

The authors have nothing to report.

## Consent

The authors have nothing to report.

## Conflicts of Interest

The authors declare no conflicts of interest.

## Supporting Information

Additional supporting information can be found online in the Supporting Information section.

## Supporting information


**Supporting Information** Table S1: Summary of key studies investigating NK‐cell alterations in adenomyosis and related conditions. The table consolidates information on study design (subjects/model, sample size), tissue and sample types, NK‐cell populations studied, key findings, and notable discrepancies across studies, organized into three sections: (A) studies directly examining adenomyosis, (B) studies on endometriosis used as a supporting analogy, and (C) cross‐condition studies. This Supporting Information supports the critical synthesis of evidence presented in Section 4.1 of the main text.

## Data Availability

The data that support the findings of this study are available from the corresponding author upon reasonable request.
