# Supplementary material for: Natural Killer Cells in Uterine Adenomyosis: Immunological Mechanisms and Therapeutic Perspectives
Source: J Immunol Res. 2026 Aug 3;2026:3833044. doi: 10.1155/jimr/3833044 (PMC13434032; doi:10.1155/jimr/3833044)
Supplement: Supplementary file 1 [file JIMR-2026-3833044-s001.docx]

**Table 1. Summary of key studies investigating NK cell alterations in adenomyosis and related conditions.**

*The table highlights the heterogeneity in study design, disease context, and reported findings, underscoring the discrepancies discussed in Section 4.1.*

| **Study (Ref)** | **Disease Focus** | **Subjects / Model** | **Sample Size (n)** | **Tissue / Sample Type** | **NK Cell Population Studied** | **Key Findings** | **Notes on Discrepancies** |
| --- | --- | --- | --- | --- | --- | --- | --- |
| **A. Studies directly examining adenomyosis** | | | | | | | |
| Bourdon et al. 2024 [17] | Adenomyosis | AM mouse model + controls | 44 (16 TAM + 28 control, across 2 experiments) | Uterine tissue (implantation sites) | uNK cells | Altered uterine immune profile incl. reduced NK cell abundance during implantation; decreased fertility | Supports numerical deficiency hypothesis in AM |
| Liu et al. 2023 [127] | Adenomyosis | AM patients vs. myoma controls (hysterectomy) | 80 AM + 62 controls = 142 total | Peripheral blood, eutopic/ectopic endometrium | CD8+ T cells (NKG2A+); pNK | Increased NKG2A+CD8+ T-cell exhaustion in AM; correlates with disease severity | One of few direct AM-patient studies; focus on T-cell exhaustion, limited NK-specific phenotyping |
| Bourdon et al. 2021 [64] | Adenomyosis | Systematic review | N/A (review) | N/A | NK cells (reviewed across studies) | Comprehensive documentation of immune dysregulation in AM; NK cell dysfunction identified as recurring theme | Review-level evidence confirming paucity of direct AM NK studies |
| Stratopoulou et al. 2023 [24] | Adenomyosis | AM hysterectomy specimens | 17 FFPE + 16 fresh biopsies (AM/non-AM split not detailed in abstract) | Eutopic/ectopic endometrial tissue | M2 macrophages (NK cells not directly assayed) | M2 macrophages enhance endometrial cell invasiveness in AM; implies altered NK-macrophage crosstalk | Indirect evidence: macrophage-related alteration without NK cell quantification |
| Niu et al. 2023 [35] | Adenomyosis | AM patients (single-cell profiling) | 1 (scRNA-seq) + 3 (RT-qPCR validation) = 4 total | Eutopic endometrium, ectopic lesions | scRNA-seq immune landscape | Immune cell composition changes including NK subset shifts; fibrosis-related remodeling in AM | scRNA-seq evidence; limited n for scRNA-seq (n=1) |
| **B. Studies on endometriosis (cited as supporting analogy)** | | | | | | | |
| Hosseinzadeh et al. 2024 [98] | Endometriosis | Advanced EM (stage III/IV) vs. controls | 13 EM + 13 controls = 26 total (11 EM + 11 controls for PF analysis) | Peripheral blood, peritoneal fluid | Local NK cells (PD-1+, TIM3+ phenotyping) | Higher PD-1+ (but not TIM3+) exhausted NK cells in peritoneal fluid of advanced EM; not in peripheral blood | Spatial heterogeneity confirmed: exhaustion restricted to lesional site |
| U. T et al. 2022 [100] | Endometriosis (pelvic) | Women with/without pelvic EM | 27 EM + 13 controls = 40 total | Peritoneal fluid (laparoscopy) | Peritoneal NK cells (chemotaxis assay) | Decreased peritoneal NK cell chemotaxis in pelvic EM; impaired trafficking to lesion site | Chemotaxis defect: suggests impaired NK recruitment, not just cytotoxicity failure |
| Li et al. 2026 [91] | Endometriosis | EM patients (scRNA-seq + GEO datasets) | 3 EM ectopic + 3 normal = 6 (scRNA-seq) + 3 GEO datasets | Eutopic/ectopic endometrium | NK cells (scRNA-seq) | Single-cell biomarkers for NK dysfunction in EM-associated immune dysregulation; transcriptional exhaustion signatures | Transcriptomic evidence for NK dysfunction; no AM-specific data |
| IL-15/EM study [92] | Endometriosis | In vitro endometrial stromal cell model | N/A (in vitro) | Endometrial stromal cells | NK cells (cytotoxicity assay) | IL-15 promotes endometrial stromal cell proliferation and inhibits NK cell killing activity | In vitro only; supports IL-15 as double-edged cytokine in EM/AM microenvironment |
| **C. Cross-condition studies (AM + EM or general reproductive immunology)** | | | | | | | |
| Shi et al. 2025 [18] | AM + EM | Review of immune microenvironment | N/A (review) | N/A | Multiple immune populations incl. NK cells | Endometrial immune microenvironment perturbations in both EM and AM; impact on reproduction and pregnancy | Key bridging review linking EM and AM immunology |
| Mai et al. 2025 [103] | Reproductive failures (RIF, RPL) | Review of uNK receptor expression (175 articles reviewed) | N/A (review) | N/A | uNK cells (NKp46 and receptor phenotyping, reviewed) | Altered NKp46 and inhibitory receptor expression on uNK cells in reproductive failure | Review-level evidence; AM not specifically studied |
| Bai et al. 2020 [114] | Endometrial diseases | Review of HLA-G/KIR2DL4 | N/A (review) | N/A | uNK cells (KIR2DL4-mediated) | HLA-G/KIR2DL4 axis regulates immune tolerance in endometrial pathology; dysregulation in EM | Mechanistic bridge to AM immune evasion hypothesis |
| Diaz-Hernandez et al. 2021 [70, 96] | Reproductive immunology | Review of uNK biology | N/A (review) | N/A | uNK cells (comprehensive) | uNK cells shift from foe to friend depending on hormonal context; progesterone-driven maturation critical | Physiological baseline for pathological uNK alterations in AM |

As summarized in Table 1, the existing literature on NK cells in AM is characterized by a striking paucity of AM-specific primary studies, with the majority of mechanistic evidence extrapolated from endometriosis research. This limitation underscores the critical need for dedicated investigations into NK cell biology within the AM microenvironment.
